# Supplementary material for: Performance of a Novel Real-Time PCR-Based Assay for Rapid Monkeypox Virus Detection in Human Samples
Source: Microorganisms. 2023 Oct 8;11(10):2513. doi: 10.3390/microorganisms11102513 (PMC10609174; doi:10.3390/microorganisms11102513)
Supplement: Supplementary file 1 [file microorganisms-11-02513-s001.zip › microorganisms-2654420-supplementary.pdf]

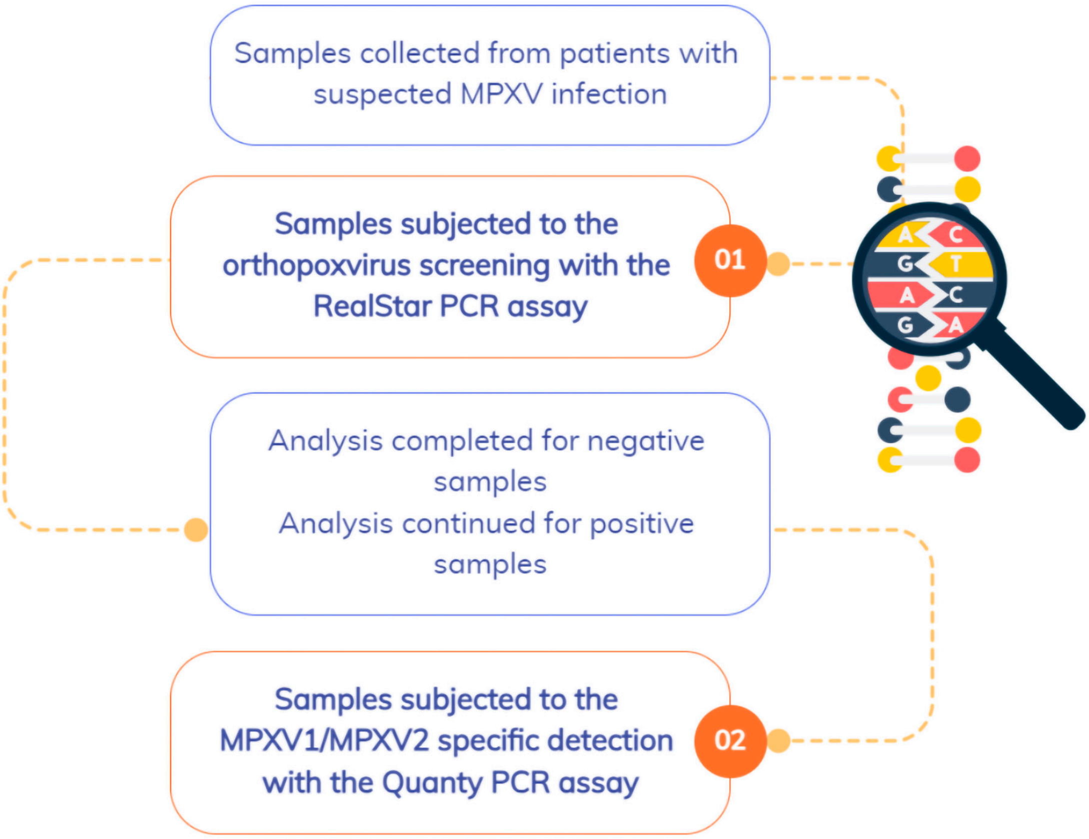

Figure S1. In-house real-time PCR based diagnostic algorithm for the detection of MPXV in clinical samples. Two steps of testing are performed, one to detect an orthopoxvirus in a sample and one to confirm a mpox virus clade I/clade II (MPXV1/MPXV2) in the positive sample from the first step. The diagnostic process, which takes a few hours to complete, has been validated using the MPXV DNA extracted from the skin lesions of an infected patient, which had been kindly provided by the Italian National Institute of Public Health.

Table S1. QIAstat-Dx assay results for samples collected at the time of diagnosis or follow-up for mpox (n = 108).

Patient #	Samples at diagnosis			Samples at first follow-up (Median [IQR]) time, 8 [7–12] days)			Samples at second follow-up (Median [IQR]) time, 17 [12–20] days)		
	**VS**	**OS**	**WB**	**VS**	**OS**	**WB**	**VS**	**OS**	**WB**
1	Pos	– [1]	Pos	Pos	Neg	Neg	–	Neg	Neg
2	Pos	Neg	–	–	–	Neg	–	–	–
3	Pos	Pos	Pos	–	Neg	Neg	–	–	–
4	Pos	Neg	Neg	–	Neg	Neg	–	–	–
5	Pos	Neg	Neg	–	Neg	Neg	–	–	–
6	Pos	Pos	Pos	Pos	Pos	Pos	–	Neg	Neg
7	Pos	Pos	Pos	–	–	–	–	–	–
8	Pos	Pos	Pos	–	Neg	Neg	–	–	–
9	–	Pos	Pos	–	–	–	–	–	–
10	Pos	Pos	Neg	–	Pos	Pos	Pos	–	Neg
11	Pos	Neg	Neg	–	Neg	Neg	–	–	–
12	Pos	Pos	Neg	–	Pos	Neg	–	–	Neg
13	Pos	Neg	Neg	Neg	–	Pos	–	–	–
14	Neg	Pos	Neg	–	–	–	–	–	–
15	Pos	Pos	Neg	–	Neg	Neg	–	–	–
16	Pos	Pos	Neg	–	Neg	Neg	–	–	–
17	Pos	Pos	Pos	Pos	Neg	Neg	Neg	–	–
18	Pos	Pos	Neg	Pos	Neg	Neg	Neg	Neg	Neg
19	Pos	Pos	Pos	–	Pos	Neg	–	Neg	Neg
20	Pos	Neg	Neg	Neg	Neg	Neg	–	–	–

[1] The symbol indicates that no (positive or negative) result was available, as no sample was obtained from the patient.
IQR, interquartile range; VS, vesicular swab; OS, oropharyngeal swab; WB, whole blood.

Table S2. Description of positive PCR results for 51clinical samples included in the study.

			Results (expressed as Ct) by each of indicated assays		
**Patient #**	**Sample #**	**Type of sample**	**Reference**	**RealStar Orthopoxvirus PCR kit 1.0**	**QIAstat-Dx Viral Vesicular panel**
1	1	Whole blood	31.0	30.2	31.2
	2	Vesicular swab	23.2	23.8	24.1
	3	Vesicular swab	24.0	23.1	24.2
2	4	Vesicular swab	37.2	36.5	38.1
3	5	Whole blood	35.4	34.6	36.1
	6	Oropharyngeal swab	23.2	22.7	24.0
	7	Vesicular swab	18.2	18.0	18.0
4	8	Vesicular swab	22.0	21.0	22.0
5	9	Vesicular swab	18.4	19.0	19.0
6	10	Whole blood	33.0	32.2	34.1
	11	Oropharyngeal swab	23.9	24.8	25.0
	12	Vesicular swab	25.4	26.0	26.0
	13	Whole blood	38.1	38.0	38.3
	14	Oropharyngeal swab	22.7	23.4	25.1
	15	Vesicular swab	17.9	18.0	19.3
7	16	Whole blood	34.0	34.0	35.1
	17	Oropharyngeal swab	25.8	22.9	26.0
	18	Vesicular swab	23.0	21.0	22.0
8	19	Whole blood	35.2	33.0	35.4
	20	Oropharyngeal swab	34.8	30.8	35.0
	21	Vesicular swab	24.4	23.0	35.0
9	22	Whole blood	37.5	38.9	38.0
	23	Oropharyngeal swab	30.1	29.2	30.0
10	24	Oropharyngeal swab	30.2	29.0	35.1
	25	Vesicular swab	20.6	19.0	21.0
	26	Whole blood	36.6	37.7	38.7
	27	Oropharyngeal swab	27.3	26.0	29.8
	28	Vesicular swab	33.1	32.0	34.2
11	29	Vesicular swab	22.9	22.0	24.0
12	30	Oropharyngeal swab	30.9	29.0	31.4
	31	Vesicular swab	32.7	33.0	33.8
	32	Oropharyngeal swab	33.5	34.0	35.8
13	33	Vesicular swab	25.8	24.0	26.6
	34	Whole blood	37.4	36.0	38.5
14	35	Oropharyngeal swab	23.0	22.0	23.1
15	36	Oropharyngeal swab	26.0	22.0	27.3
	37	Vesicular swab	22.1	20.0	22.4
16	38	Oropharyngeal swab	35.1	34.6	36.1
	39	Vesicular swab	20.7	21.0	21.0
17	40	Vesicular swab	29.0	29.0	30.0
	41	Oropharyngeal swab	24.3	21.0	23.0
	42	Whole blood	32.9	30.0	32.1
	43	Vesicular swab	34.7	32.0	35.3
18	44	Vesicular swab	28.0	27.0	30.4
	45	Oropharyngeal swab	22.3	22.0	24.1
	46	Vesicular swab	36.8	37.0	38.6
19	47	Vesicular swab	22.0	23.0	23.5
	48	Oropharyngeal swab	23.5	21.0	24.7
	49	Whole blood	30.1	32.4	33.1
	50	Oropharyngeal swab	38.7	38.0	39.0
20	51	Vesicular swab	30.0	29.1	31.8

Ct, cycle threshold.
